# The Neuroprotective Effect of Therapeutic Hypothermia in Cognitive Impairment of an Ischemia/Reperfusion Injury Mouse Model

**DOI:** 10.3390/medicina60030350

**Published:** 2024-02-20

**Authors:** Ji Sun Lim, Shin Kim, Mee-Na Park, Hyunsu Lee, Hye Suk Baek, Jin Kyung Kim, Hae Won Kim, Jeong-Ho Hong

**Affiliations:** 1 Department of Nuclear Medicine, Keimyung University Dongsan Hospital, Daegu 42601, Republic of Korea; lzsunny@hanmail.net (J.S.L.); parkmn1223@gmail.com (M.-N.P.); 2 Department of Neurology, Keimyung University Dongsan Hospital, Daegu 42601, Republic of Korea; 3 Department of Immunology, School of Medicine, Keimyung University, Daegu 42601, Republic of Korea; god98005@dsmc.or.kr (S.K.); sftwtmt@hanmail.net (H.S.B.); 4 Department of Physiology, Pusan National University School of Medicine, Yangsan 50612, Republic of Korea; hyunsu.lee@pusan.ac.kr; 5 Department of Microbiology, School of Medicine, Keimyung University, Daegu 42601, Republic of Korea; pcjlovesh6@kmu.ac.kr

**Keywords:** ischemic stroke, hypothermia, cognition, neuroprotection

## Abstract

*Background and Objectives*: Therapeutic hypothermia (TH) shows promise as an approach with neuroprotective effects, capable of reducing secondary brain damage and intracranial pressure following successful mechanical thrombectomy in the acute phase. However, its effect on cognitive impairment remains unclear. This study investigated whether TH can improve cognitive impairment in a mouse model of transient middle cerebral artery occlusion followed by reperfusion (tMCAO/R). *Materials and Methods*: Nine-week-old C57BL/6N mice (male) were randomly assigned to three groups: sham, tMCAO/R, and tMCAO/R with TH. Cognitive function was assessed 1 month after model induction using the Y-maze test, and regional cerebral glucose metabolism was measured through positron emission tomography with fluorine-18 fluorodeoxyglucose. *Results*: tMCAO/R induced cognitive impairment, which showed improvement with TH. The TH group exhibited a significant recovery in cerebral glucose metabolism in the thalamus compared to the tMCAO/R group. *Conclusions*: These findings indicate that TH may hold promise as a therapeutic strategy for alleviating ischemia/reperfusion-induced cognitive impairment.

## 1. Introduction

Mechanical thrombectomy (MT) is widely used to restore blood flow after acute intracranial artery occlusion [1]. Despite successful MT procedures leading to the restoration of blood flow, reperfusion injury may still occur, and may worsen neurological outcomes. Specifically, reperfusion injury can elevate reactive oxygen species (ROS) levels, causing direct harm to mitochondria and lipid peroxidation [2]. Excessive ROS triggers the activation of antioxidant enzymes, including superoxide dismutase, glutathione peroxidase, glutathione reductase, and catalase. Notably, glutathione, a significant antioxidant contributing to the detoxification of ROS, significantly decreases post-cerebral ischemia [3,4]. Cerebral ischemia causes reduced blood flow and diminished oxygen and glucose delivery to the brain. These conditions contribute to excitotoxicity and impaired functioning of astrocytic glutamate transporter 1 [5,6]. Astrocytes, one of the most prevalent neuroglia cell types, play a crucial role in neuronal injury. Following an ischemic stroke, astrocytes become activated, and their reactive state contributes to microglia activation, potentially causing neuronal death through various mechanisms [7].

Furthermore, immune cell activation is accompanied by the upregulation of cytokines and chemokines. Consequently, ischemia-reperfusion injury induces chronic neurological dysfunction, particularly cognitive impairment, through brain cell death. In the real clinical setting, cognitive impairment has been reported in over 80% of patients with ischemic stroke [8,9]. Ettelt et al. reported that, despite the favorable functional outcome, 86% of patients with acute large-vessel artery occlusion who underwent MT had cognitive impairment 3 months after onset [8]. These findings highlight the importance of clinicians being vigilant regarding cognitive impairment, as it strongly correlates with reduced quality of life, emphasizing the need for swift recanalization efforts.

Body temperature increases 4–6 h post-stroke, with a more pronounced rise in severe cases [10]. Given the vulnerability of the central nervous system to high temperatures, hyperthermia is closely associated with cognitive impairment [11]. Furthermore, patients with elevated body temperatures during intra- and post-ischemic periods demonstrated unfavorable outcomes post-MT [12]. Specifically, each 1 °C increase in temperature significantly increased the risk of impaired functional independence and mortality [12]. Therefore, therapeutic hypothermia (TH) has emerged as a promising strategy in the clinical field to mitigate ischemia-reperfusion injury and achieve favorable neurological outcomes. Nevertheless, most extant TH studies have primarily focused on mitigating cerebral edema, which could lead to increased intracranial pressure in the acute phase, and there remains a scarcity of investigations exploring the effect of TH on cognitive function.

Given the facts above, we hypothesized that TH could ameliorate impaired cognitive function by mitigating ischemia-reperfusion injury. To validate our hypothesis, we investigated the effects of TH on cognition using the Y-maze test. We assessed topographical changes in glucose metabolism through F-18 fluorodeoxyglucose positron emission tomography scans in a mouse model of transient middle cerebral artery occlusion.

## 2. Materials and Methods

**Transient middle cerebral artery occlusion/reperfusion (tMCAO/R) model** 

**Figure 1:**
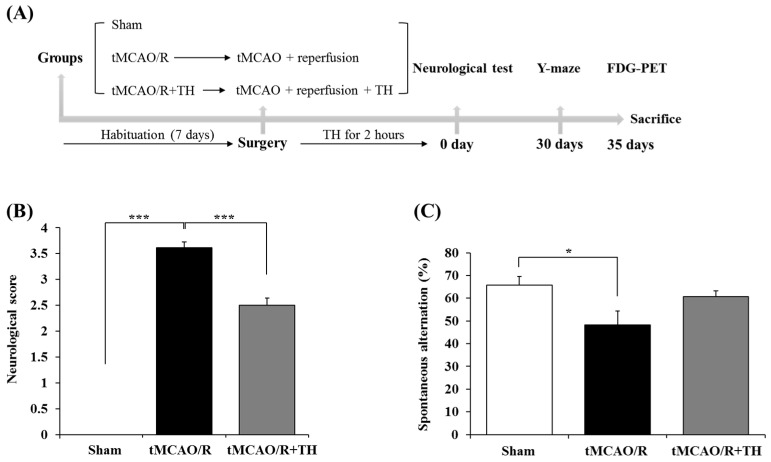
Comparison of neurological score and spatial working memory among sham, tMCAO/R, and tMCAO/R + TH groups. (**A**) Experimental schedule for behavioral study and FDG-PET using tMCAO/R mouse model. (**B**) A neurological score test was performed post-surgery. (**C**) The Y-maze test was performed 30 days post-surgery. Data were expressed as mean ± SEM, and statistical analysis was performed by Student’s *t*-test. (* *p* < 0.05, *** *p* < 0.001).

Male C57BL/6 mice of SPF-grade (9 weeks old) were obtained from Samtako Bio (Osan, Republic of Korea) and bred in a controlled environment (22 °C and 12 h light/dark cycle, lights on at 6 AM) with free access to water and food. The animals were acclimatized for 1 week before initiating tMCAO/R modeling. The mice were categorized into three groups: (1) sham (n = 13), (2) tMCAO/R (n = 18), and (3) tMCAO/R + TH (n = 14). Following induction of anesthesia with 4.0% isoflurane evaporated in a nitrous oxide (N_2_O)/oxygen (O_2_) (70:30) gas mixture, we conducted tMCAO/R or sham surgery according to the procedures outlined in a previous study. (Figure 1A) [13]. In summary, we introduced a 6–0 nylon monofilament with a silicone-coated tip (0.22 mm; Doccol Co., Sharon, MA, USA) through the external carotid artery into the right internal carotid artery, advancing it to 6 mm from the internal carotid bifurcation site. Subsequently, we temporarily covered the surgical site, and anesthesia was discontinued. After 60 min of occlusion, the mice were re-anesthetized for monofilament removal. The mice in the sham-operation group underwent an identical procedure as the tMCAO/R group, excluding the actual MCA occlusion. Figure 1A illustrates the specifications of the experimental design employed in this study. The rectal temperature of the mice was carefully maintained at 37 ± 0.5 °C during anesthesia, using an infrared lamp and a heat blanket pad. Furthermore, cerebral blood flow (CBF) was monitored throughout the experiment using laser Doppler flowmetry (Omega Flow FLO-C1 BV; Omega Wave, Tokyo, Japan). Mice showing a decrease in CBF exceeding 70% of the basal level were excluded from the analysis. All animal experiments conformed to ethical standards and received approval from the Institutional Animal Care and Use Committee of Keimyung University (IACUC No: KM-2019-09R2), in accordance with the principles delineated in the NIH Guide for the Care and Use of Laboratory Animals.

**TH** 

TH was conducted as described in a previous study [14]. A 70% alcohol spray was briefly administered to the entire mouse body, ensuring strict control of the core temperature within the range of 32–34 °C. This hypothermic state was sustained for 2 h following reperfusion, after which the body temperature gradually returned to 37 °C. [15,16]. The temperature was closely regulated throughout the TH process using a DAS-7007R (BMDS, Seaford, DE, USA) body temperature feedback system and an IPTT-300 transponder (BMDS, Seaford, DE, USA).

**Neurological function test** 

Following surgery, neurological deficits were assessed using the Bederson score (ranging from 0–4) in the sham (n = 13), tMCAO/R (n = 18), and tMCAO/R + TH (n = 14) groups [17]. We chose the Bederson scoring test because of its significant association with infarct volume across scores ranging from 0–4 [18]. Scoring was briefly based on the following criteria: 0 points for no observed deficit, 1 point for forelimb flexion, 2 points for forelimb flexion and circling, 3 points for partial paralysis on the affected side, and 4 points for no spontaneous motor activity.

**Cognitive function test** 

Cognitive function was assessed using the Y-maze test, which utilized a maze with three arms constructed from white plexiglass. Each arm was 40 cm long, 12 cm high, and 4 cm wide, and positioned 120° from each other. During the 8 min testing period, the mice were placed on one arm of the device and allowed to move freely through the maze. The behavior change (%) was calculated by dividing the number of alternations by the number of possible triads × 100 [19].

**F-18 fluorodeoxyglucose positron emission tomography (FDG-PET)** 

One month post-surgery, the survival rates for each group were as follows: (1) sham (n = 13/13), (2) tMCAO/R (n = 12/18), and (3) tMCAO/R + TH (n = 14/14). Mice underwent F-18 FDG PET to assess cerebral glucose metabolism using the Triumph II PET/CT system (Lab-PET8; Gamma Medica-Ideas, Waukesha, WI, USA). Before the PET scan, the mice underwent a 12 h fast. The mice were anesthetized with 2.0% isoflurane in a nitrous oxide/oxygen (N_2_O/O_2_, 70:30) mixture and intravenously administered approximately 7.4 MBq of F-18 FDG via the tail vein. PET scanning for whole-brain images was conducted around 30 min post-F-18 FDG injection, with the PET scan lasting for a duration of 5 min. The obtained data were assumed to show cerebral glucose metabolism. For the spatiotemporal quantification of cerebral glucose metabolism, a volume-of-interest (VOI) analysis was conducted for each scan using the PMOD software package (version 3.9; PMOD Technologies, Ltd., Zurich, Switzerland), along with a mouse brain template and atlas, as previously described, with some modifications [20,21]. PMOD was employed for the transformation of each mouse brain PET dataset into the relevant space, and an automated application of a mouse brain atlas was executed to evaluate F-18 FDG uptake. Standardized F-18 FDG uptake values within defined subregions of the mouse brain were obtained. The mouse brain VOI atlas was iteratively utilized in conjunction with a standard brain model to optimize the fusion of experimental data. The regional standardized F-18 FDG uptake values ratio (SUVR) was computed by dividing the standardized F-18 FDG uptake value for the individual target region by the corresponding region in the left cerebral hemisphere. Each group consisted of 10 mice, excluding those with unclear PET scan images or significant deviations.

**Cell culture** 

HT22 cells, derived from immortalized primary mouse hippocampal neuronal cells, are utilized in memory-related studies related to memory that focus on mature hippocampal neurons. Alterations in mitochondrial function, intracellular Ca^2+^, and pH homeostasis during hypoxic conditions can cause neuronal cell death [22,23]. HT22 cells were cultured in Dulbecco’s modified Eagle’s medium (DMEM, LM001-05, Welgene, Gyeongsan, Republic of Korea) supplemented with 10% heat-inactivated fetal bovine serum (FBS, 160,004, Gibco/Thermo Fisher Scientific, Waltham, MA, USA) and 1% penicillin-streptomycin (15,140,122, Gibco/Thermo Fisher Scientific, Waltham, MA, USA). The cells were cultured in a humidified CO_2_ incubator (Sanyo, Osaka, Japan) at 37 °C with a 5% CO_2_/95% air atmosphere, and sub-culturing was conducted every 2 days. The culture dishes were utilized to maintain aseptic conditions, preventing contamination.

**In vitro hypoxia/reoxygenation (H/R) model** 

Cells were counted using a hemocytometer and trypan blue solution (15,250,061, Gibco/Thermo Fisher Scientific, Waltham, MA, USA). HT22 cells were trypsinized and stained at approximately 80% confluence on a 100 mm plate. A total of 2.84 × 10^6^ cells/mL were counted, and then 35 μL was aliquoted per well, resulting in a final density of 1 × 10^5^ cells/well. The cells were cultured in DMEM supplemented with 10% FBS and 1%penicillin in a 5% CO_2_ humid atmosphere at 37 °C. After a 24 h period, cells in the H/R group were cultured in a serum-free and glucose-free medium within a chamber containing 0.5% O_2_ and 5% CO_2_ at 37 °C for 6 h. Glucose (A2494001, final concentration 11 mM, Gibco/Thermo Fisher Scientific, Waltham, MA, USA) was then added to the hypoxia medium, and the cells were cultured at 37 °C in a humidified 5% CO_2_ incubator for an additional 24 h. In the TH group, cells were cultured at 32 °C following the hypoxia challenge.

**Western blotting** 

HT22 cells were harvested and prepared for Western blotting. Briefly, cellular proteins were lysed with RIPA buffer (EBA-1149; ELPIS-BIOTECH, Daejeon, Republic of Korea). Total protein quantities were assessed with a BCA protein assay kit (23,225, Thermo Scientific^TM^, Waltham, MA, USA). Forty micrograms of protein samples were separated on a sodium dodecyl sulfate-polyacrylamide gel and then transferred to polyvinylidene fluoride membranes (IPVH00010, Merck Millipore Corp., Billerica, MA, USA). The proteins on the membranes were incubated with primary antibodies against poly (ADP-ribose) polymerase (PARP, #9532, 1:1000, cell signaling, Lane Danvers, MA, USA), cleaved caspase-3 (#9664, 1:1000, cell signaling) or α-tubulin (sc-5286, Santa Cruz, Dallas, TX, USA) and the corresponding secondary antibodies conjugated with horseradish peroxidase (sc-525409, #7047, 1:5000). Protein bands were visualized using SuperSignal™ West Pico Chemiluminescent Substrate (34,580, Pierce, Cheshire, UK), digitalized with FUSIONSOLO5 (KOREA BIOMICS, Seoul, Republic of Korea). Western blot band quantification was performed using the Image J program (version 1.51; https://imagej.net).

**Statistical analysis** 

Statistical analyses were conducted using SPSS software version 25.0 (SPSS Inc., Chicago, IL, USA). The comparison between the means of the two groups was assessed using Student’s *t*-test, with significance denoted by asterisks (*) for *p*-value < 0.05.

## 3. Results

### 3.1. Hypothermia Improved Functional Outcome and Cognitive Function

To assess the impact of TH on functional outcomes in the tMCAO/R group, we employed the Bederson 0–4 neurological scoring system. The functional outcomes were decreased in the tMCAO/R group and improved in the tMCAO/R + TH group (Figure 1B). Subsequently, to investigate the effect of TH on short-term memory, we conducted Y-maze behavioral tests in the tMCAO/R mouse model. The findings revealed a notable decrease in the percentage of spontaneous alterations following tMCAO/R when compared to sham controls (Figure 1C). Treatment with TH exhibited a trend towards an increased percentage of spontaneous changes in comparison to tMCAO/R without TH, although this difference did not attain statistical significance (Figure 1C). These data suggested that TH tended to improve spatial motor memory that tMCAO/R reduced.

### 3.2. Cerebral Glucose Metabolism

**Figure 2:**
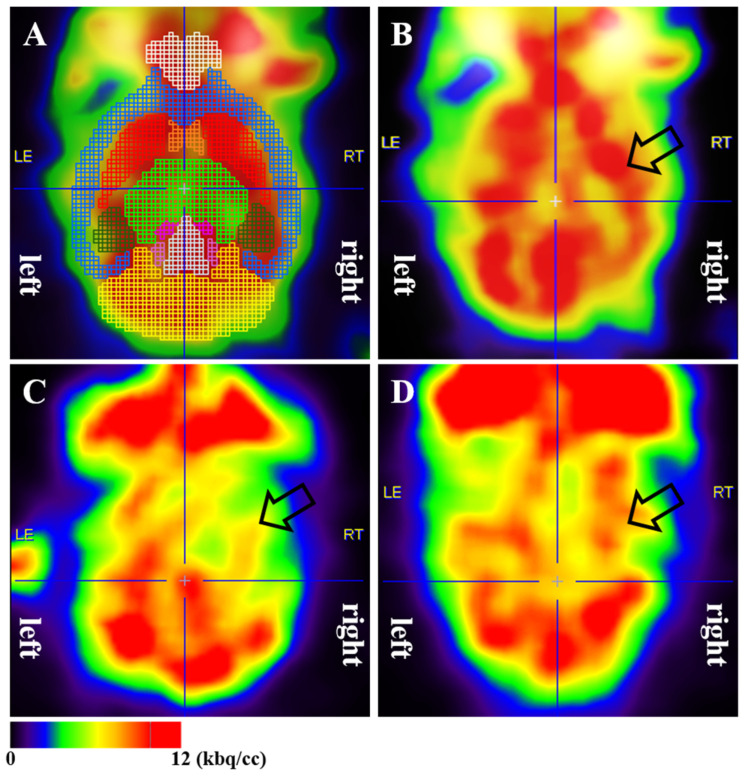
To evaluate cerebral glucose metabolism, a PET scan was performed 30 min after F-18 FDG injection using the Triumph II PET/CT system (Lab-PET8; Gamma Medica-Ideas, Waukesha, WI, USA), which has a resolution of FWHM 1.35 mm. (**A**) Regional standardized F-18 FDG uptake value ratios (SUVRs) were calculated based on a volume-of-interest analysis performed using the PMOD software package (version 3.9; PMOD Technologies, Ltd., Zurich, Switzerland) with a mouse brain template. (**B**) A sham-operated mouse shows no abnormal glucose metabolism in the right striatum. (**C**) A tMCAO/R mouse shows decreased glucose metabolism in the right striatum. (**D**) A tMCAO/R + TH mouse shows no change in glucose metabolism in the right striatum compared to a sham-operated mouse—TH upregulated glucose metabolism in the brain induced by tMCAO/R injury. The Brain PET image in the figure has been converted to rainbow colors, with black representing 0 and red representing 12 kbq/cc. The arrow indicated the location of the right striatum in the mouse brain.

To explore the impact of TH on cerebral glucose metabolism, F-18 FDG PET was conducted in the sham, tMCAO/R, and tMCAO/R + TH groups. The regional SUVRs were determined using VOI analysis (Figure 2). One month after tMCAO/R surgery, the SUVRs of the right hippocampus, left striatum, right striatum, left thalamus, right thalamus, and central gray were significantly lower in the tMCAO/R group compared to the sham group. Additionally, the SUVRs of the left thalamus were considerably higher in the tMCAO/R + TH group than in the tMCAO/R group (Table 1). However, no significant differences were observed in the SUVR in other brain regions.

### 3.3. Therapeutic Hypothermia Attenuated Hypoxia/Reoxygenation(H/R)-Induced Neuronal Apoptosis

**Figure 3:**
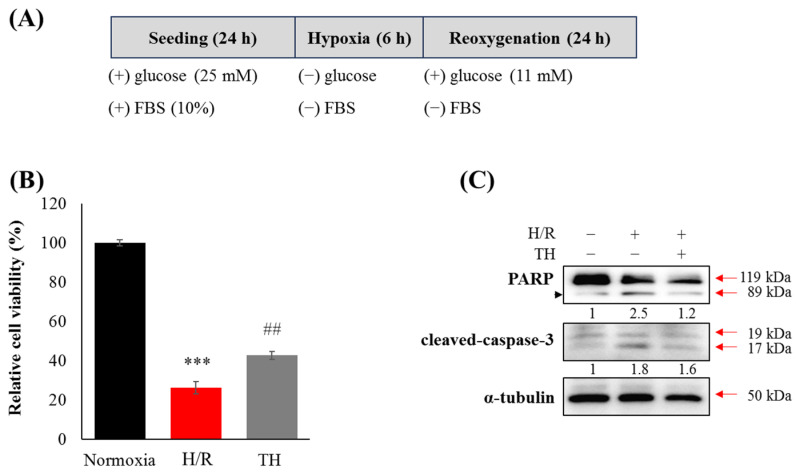
Therapeutic hypothermia attenuated H/R-induced neuronal apoptosis by modulating the expression of PARP and cleaved-caspase-3. (**A**) The experimental scheme for the in vitro hypoxia/reoxygenation model is presented. (**B**) Cell viability assay was performed following H/R with therapeutic hypothermia (TH) or without. (**C**) The expression levels of apoptotic protein markers were assessed using Western blotting. α-tubulin was used as a loading control (thick band). Proteolytic PARP cleavage was indicated by an arrowhead. Data were expressed as mean ± SEM, and statistical analysis was conducted using the Student’s *t*-test. (***; compared to normoxia, *p* < 0.001, ##; compared to H/R, *p* < 0.01).

To confirm the impact of TH on cognitive impairment in the tMCAO/R model, we established an in vitro H/R model. Following the H/R challenge, the viability of neuronal cells decreased, and PARP cleavage and cleaved-caspase-3 levels increased (Figure 3). However, TH restored the decreased neuronal viability and the expression levels of increased PARP cleavage and cleaved-caspase-3 induced by H/R.

## 4. Discussion

Ischemic stroke has been recognized as a precipitating factor in selective neuronal degeneration and neuroinflammation, contributing to consequential cognitive impairment [24]. Cognitive deficits are observed in over 70% of stroke survivors [25]. These deficits include impairments specific to the stroke lesion site, such as aphasia or memory deficits, and those arising from strategic infarcts in the hippocampus, thalamus, and key cortical regions [26]. Additionally, it includes deficits that may have preceded the onset of the stroke. Cerebral I/R injury often leads to irreversible brain damage, neuronal injury, and death, involving various complex pathological processes like oxidative stress, excitotoxicity, amino acid toxicity, release of endogenous substances, inflammation, and apoptosis [27]. Apoptotic triggers, such as Ca^2+^ accumulation and excessive ROS production induced by I/R, play a role in mitochondrial cell death [28,29]. These dysfunctions lead to the release of pro-apoptotic factors, including cytochrome *c*, endonuclease G, and apoptosis-inducing factors [30]. Pro-apoptotic factors cause apoptosome formation, converting procaspase-9 into activated caspase-9. This activated caspase-9, in turn, stimulates effector caspases like caspase-3, caspase-6, and caspase-7, which promote neuronal cell apoptosis [31].

Mild to moderate hypothermia (31–34 °C) during or after cerebral ischemia is a potent neuroprotective strategy [32,33,34,35,36,37,38]. This therapeutic approach concurrently inhibits multiple mechanisms of brain cell death and decelerates metabolic processes, effectively limiting tissue damage [36,39,40,41]. The cumulative and synergistic benefits of hypothermia offer promising clinical outcomes in patients with stroke.

**Figure 4:**
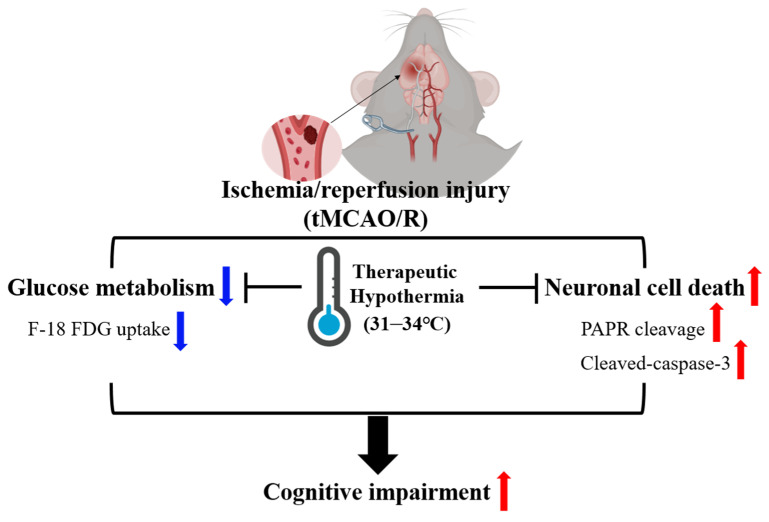
Therapeutic hypothermia mitigated I/R-induced cognitive impairment by modulating glucose metabolism and neuronal cell apoptosis. In a tMCAO/R mouse model, TH increased glucose uptake in the thalamus after I/R and reduced the protein expression of apoptosis-related markers, including PARP cleavage and cleaved-caspase-3, in a H/R hippocampal neuronal cell model.

Cerebral glucose hypometabolism is a significant feature of dementia [42]. The pattern of hypometabolism identified through F-18 FDG PET holds predictive value for the progression from baseline cognition to mild cognitive impairment [43]. Beyond its role as a biomarker, cerebral glucose hypometabolism contributes significantly to the pathophysiology of Alzheimer’s disease [44,45]. Human clinical trials targeting the restoration of cerebral bioenergetic pathways have yielded promising early results [44]. Our study demonstrated that tMCAO/R decreased glucose metabolism, and this effect improved after TH in the thalamus. Several studies have reported that cognitive dysfunction following an acute stroke is often attributed to direct injury to brain structures crucial for cognitive function, such as the thalamus or basal ganglia [25,46,47], which aligns with our results. The Y-maze test also revealed decreased short-term memory in the tMCAO/R group recovered after TH. Therefore, TH may be a potential adjuvant therapeutic option for vascular stroke. We demonstrated that TH could be effectively induced in tMCAO/R-affected injured regions, leading to neuro-regenerative effects and behavioral recovery (Figure 4).

Our study has some limitations that warrant consideration. First, the TH effect on cognitive impairment was assessed in relatively healthy young male mice. Extrapolating these findings to actual patients may encounter variations because clinical populations often comprise individuals with advanced atherosclerosis and older adult patients who have experienced ischemic stroke. Second, the therapeutic effects of hypothermia on cognitive impairment may vary depending on the degree of ischemic-reperfusion injury. The extent of injury after successful recanalization is influenced by factors such as the duration of occlusion, underlying diseases, and collateral circulation. Therefore, additional studies are essential to explore the effects of TH on cognitive function recovery in mouse models representing atherosclerosis or older adult populations. Third, our study did not comprehensively assess the damage to brain structures or isoflurane concentrations, which could influence cognitive function. For instance, the thalamus is a part of the integral neuronal network responsible for cognition [48].

Furthermore, a recent study suggested cognitive impairment occurs through the fronto–parieto–cerebellar–thalamic loop [49]. Thalamic injury has been shown to cause a decline in word retrieval, particularly concerning responses from the posterior supplementary motor area. In this study, we utilized a concentration of 4.0% isoflurane, potentially influencing cellular damage and consequently affecting cognitive function [50,51]. Finally, reactive microglia and astrocytes are observed in neurological disease states, including injury-mediated neuroinflammation, neurodegeneration, epilepsy, demyelination, and ischemic stroke [52,53]. Neuroinflammation, independently or through interactions with Aβ and tau proteins, can impair cognitive function. TH recovered cognitive impairment in an I/R mouse model and reduced neuronal cell death; however, additional investigations into TH-mediated signaling pathways, such as mitogen-activated protein kinase, nuclear factor kappa B, and Janus kinase-signal transducer and activator of transcription, are warranted. Considering these findings collectively, further studies are needed to examine thalamic damage and isoflurane concentration.

## 5. Conclusions

Our findings suggest that TH improves cognitive function in mice after tMCAO/R without causing concomitant long-term sensorimotor deficits. Additionally, TH suppressed the apoptosis of HT22 cells after H/R. Despite TH exhibiting neuroprotective effects in the tMCAO/R mouse model, further mechanistic studies are necessary to understand how TH modulates glucose metabolism while regulating neuroinflammation and neurotoxicity.

## Figures and Tables

**Table 1:** **Comparison of regional cerebral glucose metabolism after tMCAO/R surgery**.

Regions	Side	Mean (Standard Deviation)			*p*-Value		
		Sham	tMCAO/R	tMCAO/R + TH	Sham vs. tMCAO/R	Sham vs. tMCAO/R + TH	tMCAO vs. tMCAO/R + TH
Amygdala	left	0.828 (0.081)	0.904 (0.064)	0.890 (0.066)	0.115	0.213	1.000
	right	0.883 (0.107)	0.856 (0.087)	0.904 (0.088)	1.000	1.000	0.883
Cortex	left	0.960 (0.036)	0.970 (0.043)	0.943 (0.035)	1.000	1.000	0.438
	right	0.998 (0.085)	0.948 (0.107)	0.942 (0.094)	0.867	0.624	1.000
Hippocampus	left	1.141 (0.070)	1.062 (0.079)	1.151 (0.079)	0.134	1.000	0.065
	right	1.187 (0.109)	1.062 (0.078)	1.162 (0.102)	0.044 *	1.000	0.127
Midbrain	left	1.280 (0.131)	1.175 (0.086)	1.260 (0.108)	0.193	1.000	0.362
	right	1.282 (0.152)	1.143 (0.081)	1.243 (0.135)	0.106	1.000	0.345
Striatum	left	1.165 (0.078)	1.082 (0.031)	1.133 (0.069)	0.040 *	0.887	0.307
	right	1.191 (0.116)	1.074 (0.052)	1.145 (0.091)	0.043 *	0.845	0.345
Thalamus	left	1.224 (0.086)	1.102 (0.049)	1.213 (0.079)	0.007 **	1.000	0.012 *
	right	1.280 (0.136)	1.135 (0.067)	1.248 (0.113)	0.038 *	1.000	0.127
Inferior colliculi	left	1.336 (0.165)	1.205 (0.074)	0.265 (0.084)	0.083	0.570	0.850
	right	1.390 (0.209)	1.222 (0.083)	1.331 (0.124)	0.089	1.000	0.412
Superior colliculi		1.318 (0.152)	1.182 (0.053)	1.261 (0.099)	0.056	0.827	0.438
Basal forebrain		1.046 (0.041)	1.031 (0.037)	1.006 (0.042)	1.000	0.126	0.602
Central gray		1.434 (0.177)	1.237 (0.079)	0.328 (0.138)	0.023 *	0.325	0.551
Hypothalamus		1.078 (0.086)	1.043 (0.058)	1.083 (0.053)	0.899	1.000	0.675
Olfactory bulb		1.377 (0.135)	1.321 (0.173)	1.248 (0.142)	1.000	0.220	0.944

Asterisks indicate statistical significance. Regional standardized F-18 FDG uptake value ratios on the same side were compared among the sham, tMCAO/R, and tMCAO/R + TH groups using one-way ANOVA. Bonferroni post hoc analysis was employed for group comparisons, including sham and tMCAO/R, sham and tMCAO/R + TH, and tMCAO/R and tMCAO/R + TH. * *p* < 0.05, ** *p* < 0.01; All values are presented as mean (standard deviation).

## Data Availability

All data in the present study are available from the corresponding author on reasonable request.
